# Revisiting Thyroid Function in Patients Undergoing Electroconvulsive Therapy for Severe or Treatment-Resistant Depression

**DOI:** 10.3390/jcm15051740

**Published:** 2026-02-25

**Authors:** Emre Mutlu, Adile Begüm Bahçecioğlu, Şeref Can Gürel

**Affiliations:** 1 Department of Psychiatry, School of Medicine, Hacettepe University, 06230 Ankara, Türkiye; scangurel@gmail.com; 2 Department of Endocrinology and Metabolism, Gülhane Training and Research Hospital, 06010 Ankara, Türkiye; begumbahceci@hotmail.com

**Keywords:** euthyroid sick syndrome, mood disorders, thyroid hormones, unsupervised clustering

## Abstract

**Background/Objectives**: Evidence regarding the relationship between thyroid function tests (TFTs) and severe or treatment-resistant depression in euthyroid individuals remains limited. We aimed to investigate thyroid function tests (TFTs) in euthyroid patients with depression undergoing electroconvulsive therapy (ECT), evaluate associations with ECT response and depression severity, and explore whether clinically meaningful subgroups with differential thyroid function patterns can be identified. **Methods**: In this retrospective cohort study, we screened 107 inpatients who received ECT for severe or treatment-resistant depression (major depressive disorder [MDD] or bipolar disorder [BD]). Seventy-six euthyroid patients were analyzed. Clinical data, Hamilton Depression Rating Scale (HAMD) scores, and TFTs (TSH, free-T3, and free-T4) were assessed. Logistic regression, multiple linear regression and unsupervised hierarchical cluster analyses were performed. The cluster analysis used clinical and demographic variables, excluding TFTs to avoid circularity and allow thyroid parameters to be examined as secondary biological correlates. **Results**: The TFT results were not significantly associated with ECT response in euthyroid patients. The multiple linear regression revealed that the baseline HAMD scores were positively associated with free-T4 (*β* = 0.797, *p* = 0.001). Hierarchical clustering identified two subgroups; one group characterized by male sex, psychotic features, and MDD diagnosis exhibited lower TSH levels (2.12 vs. 1.49 mlU/L, Cohen’s *d* = 0.56) despite similar ECT response rates. **Conclusions**: Subtle TFT variations were not associated with ECT response but were related to depression severity and clinical phenotypes. These findings suggest that normal-range thyroid hormone variability may reflect state-related neuroendocrine patterns rather than predictors of treatment outcome. Our results should be regarded as hypothesis-generating and underline the need for prospective studies to clarify the clinical significance of thyroid function variability in severe depression.

## 1. Introduction

There has been ongoing debate about the relationship between mood disorders and thyroid dysfunction. The prevailing view suggests a bidirectional relationship, where mood disorders are associated with increased risk or severity of thyroid dysfunction, and vice versa. Depressive episodes, whether in major depressive disorder or bipolar disorder, may be related to alterations in serum thyrotropin (TSH) levels [1,2], and both overt hypo- and hyperthyroidism may present with depressive-like symptoms [3,4]. However, controversial results emerged from a population-based study and meta-analyses [5,6,7]. In addition, evidence based on symptom scales in samples of the general population is unclear regarding biochemically euthyroid states [6,8], although two recent reports described a significant relationship between depressive symptom severity and levels of TSH and free-thyroxine (FT4) in population-based cohorts that included individuals both with and without mood disorders [2,9].

**Figure 1:**
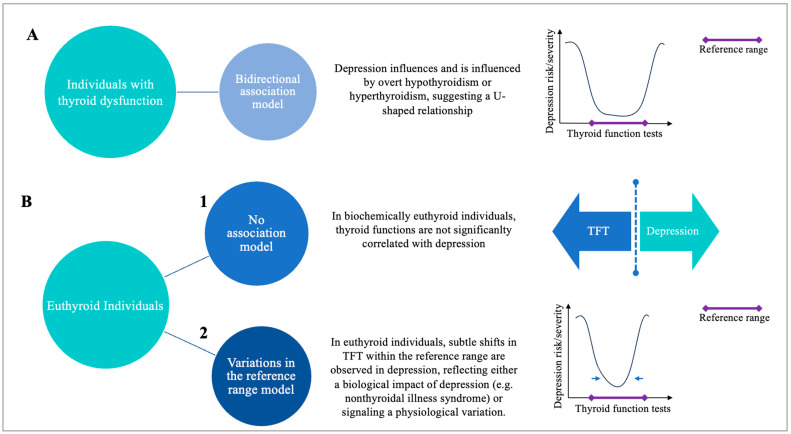
Conceptual models for the relationship between thyroid function and depression. TFTs: thyroid function tests.

Several conceptual models may be proposed to explain the relationship between depression and thyroid function (Figure 1A,B):

A. In patients with overt or subclinical thyroid dysfunction (Figure 1A): Bidirectional association model (including a U-shaped pattern): Mood disorders—particularly depression—may influence and be influenced by overt thyroid dysfunction (hypothyroidism or hyperthyroidism), reflecting a bidirectional relationship. Notably, some studies suggest a U-shaped association, whereby depressive symptoms are associated with both overt and subclinical thyroid dysfunction at the low and high extremes of TFTs [10]. Regarding the U-shaped relationship, evidence supports the role of thyroid hormones in mood regulation and the management of depression in patients with thyroid disorders [1,10,11,12,13,14,15].

B. In biochemically euthyroid individuals (Figure 1B):

1. No-association model: Several studies have failed to find a consistent relationship between thyroid hormone levels and depressive symptoms in euthyroid individuals [6,16].

2. Variations in the reference range model: In this view, subtle shifts in TSH within the reference range may either reflect the biological impact of depression—potentially mediated by hypothalamic-pituitary axis suppression due to chronic stress or illness severity, as seen in nonthyroidal illness syndrome—or signal a physiological variation that potentially predisposes individuals to severe or treatment-resistant forms of depression.

Studies involving clinically euthyroid populations have yielded mixed results. Certain studies reported no cross-sectional or longitudinal associations between thyroid function tests (TFTs) and depressive symptoms based on scales [6,16] or major or bipolar depression [5,17], suggesting a no-association model. Positive results are potentially limited by methodological issues such as the heterogeneity of the samples and using symptom scales without diagnostic evaluations [2,9,16]. Conversely, it was proposed that a group of patients with treatment-resistant depression and high-normal TSH levels (2.5–4.5 μIU/mL) may require thyroid hormone supplementation [18]. The high-normal TSH levels may correlate with poor response to antidepressant treatments [18,19,20]. Nonetheless, the clinical significance of normal-range TFTs in euthyroid patients with depression remains uncertain. Furthermore, baseline lower TSH, blunted TSH response to thyrotropin-releasing hormone, and alterations in circadian variations in TSH are well-known abnormalities in severe depression [12,21,22], suggesting variations in the reference range model.

Overall, the current evidence on the relationship between TFTs and depression is still unresolved in euthyroid patients with severe depression. Given these limitations, utilizing data-driven clustering analysis may reduce sample heterogeneity, facilitating simultaneous investigation of model B1 and B2. Thus, this study aimed to investigate thyroid functions in euthyroid patients undergoing electroconvulsive therapy (ECT) for severe or treatment-resistant depression, evaluate the relationship between TFTs and clinical features, and explore whether clinically meaningful subgroups with differential thyroid function patterns could be identified using a data-driven approach. Our hypotheses were as follows: (1) euthyroid patients with severe or treatment-resistant depression show a TSH distribution similar to the general population; (2) thyroid function tests are unrelated to response status and depression severity in euthyroid patients; (3) using unsupervised hierarchical cluster analysis, clinically distinct subgroups may emerge, within which thyroid function variability could be examined as a secondary biological correlate rather than a defining feature.

## 2. Materials and Methods

### 2.1. Participants

Participants were inpatients aged 18 years or older, receiving ECT for a depressive episode of bipolar disorder or major depressive disorder (based on DSM-IV-TR criteria) [23] between January 2010 and June 2018 at Hacettepe University Psychiatry Department. This study was planned in 2020. The study period was selected based on the availability of consistent electronic medical records and laboratory testing protocols at our institution. To ensure data homogeneity and avoid potential confounding effects arising from the COVID-19 pandemic, newer data were not included in this specific analysis. Patients’ medical charts and electronic hospitalization records were retrospectively reviewed, and diagnoses were extracted from clinical records. Exclusion criteria included neurological disorders, thyroid diseases, baseline abnormal TSH levels, ECT indications other than depressive episodes (e.g., mixed/manic episodes, catatonia), pregnancy, breastfeeding, missing Hamilton Depression Rating Scale (HAMD) scores, missing thyroid hormone measurements, and ongoing thyroid hormone treatment (L-triiodothyronine or L-thyroxine). A history of thyroid disease (hypothyroidism, hyperthyroidism, goiter, or previous thyroid surgery), abnormal thyroid function tests, or current use of L-triiodothyronine or L-thyroxine were used as exclusion criteria to ensure the inclusion of euthyroid individuals and minimize potential confounding. The final sample partially overlapped with our previous study detailed elsewhere [24]. The flow diagram of participants is represented in Figure S1. The study adhered to the latest version of the Declaration of Helsinki. All procedures were approved by the Ethics Committee of Health Sciences at Hacettepe University (approval number: GO20/533). Patient consent was waived because participants’ data were obtained anonymously, without accessible personally identifiable information, and clinical records were reviewed retrospectively by the researchers.

### 2.2. Assessment of Clinical Characteristics

Following a chart review, age, sex, psychotic features, suicidality, the number of affective episodes, the duration of hospitalization, the duration of the index episode, unsuccessful treatment trials during the episode, and the number of ECT sessions were obtained. “Suicidality” was defined as any documented suicidal ideation, intention, plan, or thoughts of death as noted in the mental status examination or patients’ risk management plans. Unsuccessful treatment trials included antidepressant switches, adding/changing augmentation strategies such as a combination of antidepressants with different mechanisms of action, adding an antipsychotic, a mood stabilizer, or triiodothyronine used for at least four weeks without clinical improvement before initiating ECT.

Indications for ECT were classified as either treatment resistance or severe depression. Treatment resistance was defined as the failure to respond to at least two adequately conducted treatment trials, described above as unsuccessful treatment trials. Severe depression was defined as the presence of psychotic features, suicidality, or a history of positive response to ECT alone in previous depressive episodes.

Seventeen-item HAMD [25,26] scores were recorded before and after ECT. Electroconvulsive therapy responders were identified by achieving a reduction of ≥60% in baseline HAMD score. The raters were trained psychiatry residents who were experienced in the use of the Turkish version of the HAMD scale. All HAMD interviews were conducted and scored by the same rater, per case.

### 2.3. Thyroid Function Tests

To measure TSH, FT4, and free-triiodothyronine (FT3) levels, early morning blood samples were collected from each patient due to diurnal variations. Blood samples were obtained following overnight fasting (~10 h). Measurements of TSH, FT4 and FT3 were analyzed after centrifugation to obtain clear serum. Serum levels of TSH (reference range: 0.38–5.33 mlU/L), FT4 (7.86–14.41 pmol/L), and FT3 (3.8–6.0 pmol/L) were measured using electrochemiluminescence immunoassay (Beckman Coulter GmbH, Brea, CA, USA).

### 2.4. Electroconvulsive Therapy Procedure

Electroconvulsive therapy was administered twice a week using the SpECTrum 5000Q (Mecta, Tualatin, OR, USA). Bilateral temporal electrode placement and brief pulse settings were applied. Seizure thresholds were determined in the first session according to Weiner and colleagues [27]. The subsequent sessions were performed at 1.5 times the seizure threshold. A minimum seizure duration of 20 s measured by using the cuff method or 25 s of seizure activity on an electroencephalogram was defined as an adequate seizure. All ECT sessions were performed by an experienced multidisciplinary team, including psychiatrists, anesthesiologists, anesthetic technicians and nurses in the department’s Psychiatric Intervention Unit.

### 2.5. Statistical Analyses

All statistical analyses were performed using IBM SPSS Statistics version 25.0 (IBM Corp., Armonk, NY, USA), R language (v.4.1.1) and ‘cluster’ package (v.2.1.6) (RCoreTeam: Vienna, Austria) [28,29]. Descriptive statistics were presented as frequencies and percentages for categorical variables and as medians with interquartile ranges (IQRs) for continuous variables due to non-normal distributions. Group comparisons for categorical variables were performed using the Chi-square test or Fisher’s exact test, while the Mann–Whitney U test was used for continuous variables. Spearman correlation coefficients were calculated to examine the correlations between TFTs and clinical features, as the data included non-normally distributed and categorical variables.

For response status, variables with a *p*-value less than 0.25 in the univariate analysis were included in the logistic regression model [30]. The Enter method was applied to identify independent risk factors associated with treatment response. Odds ratios (ORs) and corresponding 95% confidence intervals (CIs) were calculated. Model calibration was assessed using the Hosmer–Lemeshow goodness-of-fit test. In addition, multiple linear regression models with backward eliminations were conducted for examining the relationships between TFTs, depression severity—measured according to HAMD score before ECT—and the mean percentage change in HAMD score. Independent variables were selected based on the literature, including age, sex diagnosis (bipolar disorder or major depressive disorder), psychotic features, suicidality, the duration of the index episode, unsuccessful treatment trials during the episode, lithium use, and TSH, FT3, and FT4 levels.

Additionally, a hierarchical cluster analysis was conducted using a dissimilarity matrix incorporating response status, diagnosis, psychotic features, sex, and age to explore potential patient subgroups. Variables were chosen a priori based on the literature linking thyroid physiology and depressive phenotype. Thyroid hormone parameters were not included in the cluster analysis to avoid circularity, since they would subsequently be compared across subclusters. Comparisons between clusters were performed using Chi-square tests for categorical variables and Student’s *t*-tests for continuous variables. A two-tailed *p*-value of <0.05 was considered statistically significant.

## 3. Results

Out of 107 patients who underwent ECT for a depressive episode, 76 patients (*n* = 21, 28% bipolar disorder; *n* = 55, 72% major depressive disorder) were included in the analysis (Figure S1). The majority of the sample were female (60%). Psychotic features and suicidality were detected in 29% and 51%, respectively. The median age was 57 (interquartile range [IQR] = 35), the median number of previous depressive and total affective episodes was 2 (IQR = 2 and 3, respectively), and the median number of manic episodes was 0 (IQR = 1). Sixty-three patients (83%) were treated with ECT due to treatment resistance, while thirteen (17%) received ECT for severe depression alone. The two indication groups did not differ in terms of TFTs (for TSH: U = 392.000, *p* = 0.809; for FT4 U = 397.500, *p* = 0.869; for FT3 U = 326.500, *p* = 0.252). The TFT results were similar between patients with bipolar disorder and major depressive disorder (TSH U = 529.000, *p* = 0.574; FT4 U = 563.500, *p* = 0.871, for FT3 U = 531.000, *p* = 0.589). Further patient characteristics are presented in Table 1.

Sixty-one patients (80%) were classified as responders, and fifteen (20%) as non-responders. There were no significant demographic or clinical differences between responders and non-responders (Table 1). Variables with a *p*-value less than 0.25, such as age, suicidality, total days of hospitalization, the number of unsuccessful treatment trials, HAMD score before ECT, and free-T4 levels, were included in a logistic regression model. None of the variables were associated with response (Table S1).

The distribution of TSH levels was right-skewed (skewness: 1.152), with a median of 1.54 mlU/L (IQR = 1.32) (Figure S2). A correlation matrix including TSH, FT3, FT4, sex, age, diagnosis, psychotic features, suicidality, HAMD scores before and after ECT, percentage change in HAMD, the number of ECT sessions, and the duration of the index episode is presented in Table S2. TSH levels showed no significant correlations with clinical variables. FT3 was negatively correlated with age and psychotic features, but positively correlated with suicidality. FT4 was positively correlated with baseline HAMD scores prior to ECT.

We employed a multiple linear regression model to examine the relationship between baseline HAMD score (before ECT) and age, sex, diagnosis (bipolar disorder or major depressive disorder), psychotic features, suicidality, the duration of the index episode, the number of unsuccessful treatment trials during the episode, lithium use, and TSH, FT3, and FT4 levels. The regression analysis showed a significant association between baseline HAMD score and FT4 (Table 2). When the mean percentage change in the HAMD score was defined as the dependent variable, the regression analysis revealed that there was a significant association between the percentage change in HAMD score and the total number of unsuccessful treatment trials (Table 2). There were no significant associations between the percentage change in HAMD score and TSH, FT3, or FT4.

We performed hierarchical clustering using a dissimilarity matrix that included response status, diagnosis, psychotic features, sex and age. Thyrotropin and FT3 and FT4 levels were not included in the cluster analysis since they were subsequently compared between the clusters. The dendrogram for patient clustering revealed two clusters, Cluster 1 (*n* = 47) and Cluster 2 (*n* = 29) (Figure S3). Cluster 2 had higher frequencies of male sex (*n* = 22, 76% vs. *n* = 18, 17%, *χ*^2^ = 25.99, *p* < 0.001) and greater psychotic features (*n* = 17, 59% vs. *n* = 5, 11%, *χ*^2^ = 20.08, *p* < 0.001) than Cluster 1. All patients in Cluster 2 were diagnosed with major depressive disorder (*n* = 29) while half of the patients in Cluster 1 were diagnosed with bipolar disorder (*n* = 21, 45% vs. *n* = 0, *χ*^2^ = 17.91, *p* < 0.001). Cluster 1 and 2 did not differ in age (mean (sd), 50 (18) vs. 59 (19) years, respectively, *t* = −1.190, *p* = 0.060) and response rate (*n* = 37, 79% vs. *n* = 24, 83%, respectively, *χ*^2^ = 0.18, *p* = 0.668).

Group comparisons of the two clusters showed that Cluster 2 demonstrated significantly lower TSH levels than Cluster 1 (Table 3). Lithium use was more frequent in Cluster 1, while the two clusters were similar regarding suicidality, the number of ECT sessions, the number of unsuccessful treatment trials, and FT3 and FT4 levels.

## 4. Discussion

In this study, we found that the distribution of TSH levels in euthyroid patients with severe or treatment-resistant depression was right-skewed, with a median of 1.54 mIU/L, similar to the general population [31]. Response status showed no significant associations with TFTs. However, baseline depression severity was significantly associated with higher FT4 levels. Although FT3 was correlated with psychotic features and suicidality in the correlation analyses, there were no significant associations between FT3 levels and ECT response and depression severity in the regression analyses. Hierarchical clustering revealed a subgroup of patients characterized by male sex, psychotic features, and a diagnosis of major depressive disorder who demonstrated significantly lower TSH levels. However, the frequency of lithium use in this subgroup was lower than in the other group.

Rather than supporting a direct causal role of thyroid hormones in determining treatment response, our findings suggest that subtle variations in thyroid function tests within the reference range may reflect distinct clinical–neuroendocrine patterns associated with depressive phenotypes undergoing electroconvulsive therapy. In this carefully selected euthyroid sample, thyroid parameters were not associated with ECT response status, indicating that minor thyroid hormone fluctuations are unlikely to serve as predictors of ECT outcome in severe or treatment-resistant depression. This observation is clinically relevant, as it argues against overinterpreting normal-range thyroid function tests when evaluating treatment resistance in routine psychiatric practice.

Reduced TSH levels relative to controls have frequently been reported among patients receiving ECT or experiencing severe depression [12,32,33]. However, these studies had limited sample sizes, typically with fewer than 50 individuals per clinical group. Population-based cohort studies have yielded controversial findings. For instance, two large-scale population-based studies demonstrated more depressive symptoms in subjects with lower TSH levels within the normal range [8,9]. Duenas et al. reported a β value of 0.12 (95% CI −0.19 to −0.05, adjusted for age and sex) per one-unit change in natural log-transformed TSH [9], while Medici et al. found an OR of 2.2 (95% CI 1.18 to 4.17) for higher scores of depression among patients with the lowest TSH levels (0.3–1.0 mIU/L) [8]. However, these studies included heterogeneous samples with hyperthyroidism or hypothyroidism or those positive for thyroid autoantibodies. Additionally, the cohorts primarily included patients with mild to moderate depressive symptoms and lacked rigorous diagnostic evaluations for depression at baseline assessments, relying instead on clinical scales. This approach introduces a limitation since elevated symptom scores at baseline do not necessarily equate to a clinical diagnosis of depression.

In contrast, by restricting the sample to biochemically euthyroid patients undergoing ECT and excluding individuals with known thyroid disease, our findings provide evidence that thyroid function variability within the reference range is not directly related to ECT response. Instead, the observed association between higher FT4 levels and baseline depression severity may reflect broader neuroendocrine adaptations associated with illness burden rather than a specific thyroid-driven mechanism.

Our findings in terms of TSH were in line with those from population-based studies conducted by Forbes et al. and van de Ven et al., which reported no significant associations between TSH levels and current or longitudinal depression [6,16]. Additionally, a meta-analysis by Liu et al., involving 2942 patients with bipolar disorder, found similar TSH levels between depressive episode patients and healthy controls [5]. However, certain clinical variables, including trait and state characteristics of depression, were neglected in these studies. To the best of our knowledge, only two studies have specifically examined relationships among neuroticism, suicidal tendencies, and TSH levels in patients with depression [16,34]. While van de Ven et al. reported no significant relationship between TSH and neuroticism scores [16], Joffe and Levitt found a higher suicide risk in patients with low-normal TSH levels compared to those with high-normal TSH levels [34]. Using hierarchical cluster analysis, our findings suggest that demographic and clinical variables such as male sex, psychotic features, and major depressive disorder may be associated with lower TSH levels. Importantly, thyroid hormone parameters were intentionally excluded from the clustering algorithm to avoid circularity, strengthening the interpretation that differences in TSH emerged as secondary biological correlates of clinical characteristics rather than drivers of cluster formation.

Our sample included both younger and older adults. The correlation analysis showed that age was inversely correlated with FT3, while no correlation was found with TSH and FT4 (Table S2). Although the most consistent finding is elevated TSH levels in older adults, there are conflicting results regarding age-related TFT variations [35]. Regarding the relationship between age and depressive symptoms, age was negatively correlated with suicidality and positively correlated with psychotic features (Table S2). When age, suicidality, and psychotic features were adjusted for in the regression analyses, there was no significant association between the TFT, response status, and clinical improvement. However, positive longitudinal outcomes have been reported in the literature, such as a lower risk of stroke with higher TSH levels [36], better functional mobility with low-normal FT4 [37], and a decreased risk of phenotypic age acceleration with higher FT3 [38]. We are unable to make further comments due to the absence of longitudinal data. Longitudinal studies are needed to examine the clinical implications of the interplay between age, TFT variation, and depressive symptoms.

An additional point of interest in our findings is the older age in Cluster 2, which—although not reaching statistical significance (*p* = 0.060)—may carry physiological relevance. TSH levels are generally expected to increase modestly with age due to alterations in hypothalamic–pituitary–thyroid axis sensitivity and set-point regulation. However, despite this trend toward older age, patients in Cluster 2 exhibited significantly lower TSH levels compared to Cluster 1. This inverse pattern may indicate state-dependent suppression of the hypothalamic–pituitary–thyroid axis, potentially reflecting stress-related neuroendocrine adaptations as observed in severe psychiatric illness. One plausible interpretation is a pattern consistent with features of nonthyroidal illness syndrome as described in both medical and psychiatric populations. However, given the cross-sectional and retrospective design, such interpretations remain speculative and should be considered hypothesis-generating rather than mechanistic conclusions.

In our sample, the primary indication for ECT was severe or treatment-resistant depression with a median of three unsuccessful treatment trials. Previous reports suggest that patients with subclinical hypothyroidism or high-normal TSH levels may be more likely to exhibit resistance to antidepressant treatment [18]. In our sample, TSH levels were comparable between ECT responders and non-responders, providing no evidence for a direct association between TSH status and clinical response to ECT. In addition, there were no associations between the mean percentage change in HAMD score and TFTs in linear regression model 2. One possible explanation for this discrepancy is that patients with high-normal TSH levels may have been underrepresented, as only 20% of our sample had TSH concentrations exceeding 2.5 mIU/L—the threshold used by Cohen et al. to define high-normal levels. This limited distribution range could have reduced the statistical power to detect meaningful differences related to elevated TSH levels. Alternatively, the robust antidepressant effects of ECT, particularly in pharmacoresistant cases, may override the subtle modulatory effects of thyroid hormone variations that could influence response to conventional pharmacotherapy. These possibilities underscore the importance of considering sample composition and treatment modality when interpreting associations between thyroid function and clinical outcomes in depression.

Several limitations should be considered when interpreting our findings. The retrospective design, absence of longitudinal data on thyroid hormones, and small sample size constitute the major limitations. The small sample size might have limited the statistical power, potentially affecting model stability and increasing the risk of type II errors in group comparisons. Additionally, the number of predictors analyzed relative to the sample size may have increased the risk of overfitting. Therefore, while these multivariate models provide insights into potential associations, they should be interpreted as exploratory, and the stability of the identified predictors warrants validation in larger cohorts. In our study, we were not able to assess thyroid autoantibodies since there were no clinical indications for such testing among the included patients. Finally, while we excluded patients with known thyroid diseases and overt thyroid dysfunctions, we did not screen participants using thyroid ultrasonography, potentially overlooking clinical conditions such as thyroid nodules or neoplasms. However, by reducing sample heterogeneity through strict exclusion of thyroid disease and applying unsupervised clustering to identify clinically meaningful subgroups, this study provides a focused clinical framework for future research. Prospective studies incorporating longitudinal thyroid profiling, circadian hormone assessments, and complementary biological markers are needed to clarify whether observed thyroid function patterns represent transient illness-related adaptations or stable trait-like characteristics of specific depressive phenotypes.

## 5. Conclusions

The distribution of TSH levels among euthyroid patients with depression resembled that of the general population. There were no significant associations between TSH levels and ECT response, suggesting that thyroid function tests within the reference range are unlikely to serve as reliable predictors of treatment outcome in this clinical context. These findings support the view that minor thyroid hormone fluctuations should not be overinterpreted when evaluating resistance to ECT in patients without overt thyroid dysfunction. However, higher FT4 levels were associated with greater baseline depression severity, and a clinically distinct subgroup characterized by male sex, psychotic features, and a diagnosis of major depressive disorder exhibited lower TSH levels despite similar treatment response rates. Rather than implying a direct mechanistic role of thyroid hormones, these patterns may reflect state-related neuroendocrine adaptations associated with depressive phenotypes, potentially consistent with features of nonthyroidal illness syndrome.

These observations should be regarded as hypothesis-generating rather than confirmatory. The retrospective and cross-sectional design precludes causal inference, and future prospective studies incorporating longitudinal thyroid profiling and hypothalamic–pituitary–thyroid axis activity are needed to determine whether thyroid function variability within the euthyroid range represents a transient illness-related phenomenon or a stable trait associated with specific depressive subtypes. Clarifying this distinction may improve our understanding of neuroendocrine regulation in severe depression and inform future biologically informed stratification approaches.

## Figures and Tables

**Table 1:** Demographic and clinical characteristics of responders vs. non-responders.

	Total Sample *n* = 76 (%)	Responders *n* = 61 (%)	Non-Responders *n* = 15 (%)	*χ* ^2^	*p*
Sex, female	46 (60)	36 (59)	10 (67)	0.295	0.587
Diagnosis, bipolar disorder	21 (28)	18 (30)	3 (20)	Fisher’s exact	0.538
Psychotic features	22 (29)	18 (30)	4 (27)	Fisher’s exact	1.00
Suicide risk	39 (51)	29 (48)	10 (67)	1.763	0.184
Lithium users	25 (33)	20 (33)	5 (33)	Fisher’s exact	1.00
	Median (IQR)	Median (IQR)	Median (IQR)	U	*p*
Age	57 (35)	58 (32)	50 (39)	351.5	0.166
Number of previous depressive episodes	2 (2)	2 (2)	3 (2)	410.0	0.528
Number of previous manic episodes	0 (1)	0 (1)	0 (0)	426.5	0.607
Number of total affective episodes	2 (3)	2 (3)	3 (3)	441.0	0.827
Total duration of hospitalization, days	53 (32)	51 (33)	64 (42)	311.5	0.057
Duration of the index episode, weeks	31.2 (49.5)	29 (47)	33 (62)	406.0	0.501
Number of unsuccessful treatment trials	3 (2)	3 (2)	3 (2)	335.5	0.102
HAMD score before ECT	23 (10)	23 (10)	20 (10)	300.0	0.084
TSH, mlU/L	1.54 (1.32)	1.54 (1.35)	1.62 (1.19)	386.5	0.354
Free-T3, pmol/L	4.54 (1.04)	4.54 (0.98)	4.48 (1.36)	437.5	0.794
Free-T4, pmol/L	12.12 (4.20)	12.29 (4.36)	10.84 (4.25)	361.0	0.208
Number of ECT sessions	10 (5)	10 (5)	11 (5)	384.0	0.334

*χ*^2^: Chi-square test; U: Mann–Whitney U test; IQR: interquartile range; ECT: electroconvulsive therapy; HAMD: Hamilton Depression Rating Scale; TSH: thyrotropin; Free-T3: free triiodothyronine; Free-T4: free thyroxine.

**Table 2:** Multiple linear regression models for depression scale score before ECT, and mean percentage change in depression score.

Model 1 * Dependent Variable: HAMD Score Before ECT	Unstandardized Coefficients		Standardized Coefficients Beta	t	*p*	95% CI	VIF
	*β*	Std. Error					
Constant	13.872	2.943		4.713	<0.001	8.008–19.737	
Free-T4	0.797	0.232	0.371	3.439	0.001	0.335–1.259	1.000
*R* = 0.371, *R*^2^ = 0.138, *F* = 11.825; *p* = 0.001							
Model 2 * Dependent variable: mean percentage change in HAMD scores							
Constant	0.871	0.055		15.922	<0.001	0.762–0.980	
Number of unsuccessful treatment trials	−0.044	0.016	−0.314	−2.842	0.006	−0.076–(−0.013)	1.000
*R* = 0.314, *R*^2^ = 0.098, *F* = 8.074; *p* = 0.006							

* Variables that entered the model with the backward elimination method: age, sex, diagnosis of bipolar disorder or major depressive disorder, psychotic features, suicidality, duration of the index episode, unsuccessful treatment trials during the episode, lithium use, and TSH, FT3, and FT4 levels. ECT: electroconvulsive therapy; HAMD: Hamilton Depression Rating Scale; Free-T4: free thyroxine; VIF: variance inflation factor; CI: confidence interval.

**Table 3:** Comparison of two clusters.

	Cluster 1 *n* = 47	Cluster 2 *n* = 29	*χ*^2^ or *t*	*p*
Suicide risk, *n* %	27, 57%	12, 41%	1.85	0.173
Number of ECT sessions, mean (sd)	10 (4)	11 (3)	0.357	0.722
Number of unsuccessful treatment trials, mean (sd)	3 (2)	3 (2)	0.540	0.591
Lithium users, *n* %	20, 43%	5, 17%	5.205	0.023
TSH, mlU/L, mean (sd)	2.12 (1.31)	1.49 (0.89)	2.501	0.015 *
Free-T3, pmol/L, mean (sd)	4.58 (0.73)	4.48 (0.95)	0.523	0.602
Free-T4, pmol/L, mean (sd)	12.06 (3.34)	12.65 (2.97)	−0.777	0.440

*χ*^2^: Chi-square test; *t*: Student’s *t* test; ECT: electroconvulsive therapy; TSH: thyrotropin; Free-T3: free triiodothyronine; Free-T4: free thyroxine; * Cohen’s *d* = 0.56.

## Data Availability

The data presented in this study are available on request from the corresponding author. The data are not publicly available due to ethical restrictions.

## Supplementary Materials

The following supporting information can be downloaded at: https://www.mdpi.com/article/10.3390/jcm15051740/s1: Figure S1: Flow diagram of the sample. ECT: electroconvulsive therapy; TSH: thyrotropin. Figure S2: Distribution of thyrotropin (TSH) levels among sample. Figure S3: Dendrogram for patient clustering according to response status, diagnosis, psychotic features, sex and age. Table S1: Logistic regression analysis, dependent variable: response. Table S2: Correlation matrix (n = 76).

## Abbreviations

The following abbreviations are used in this manuscript:

TFTs	Thyroid function tests
ECT	Electroconvulsive therapy
MDD	Major depressive disorder
BD	Bipolar disorder
HAMD	Hamilton Depression Rating Scale
TSH	Thyrotropin
FT4	Free-thyroxine
FT3	Free-triiodothyronine
IQR	Interquartile ranges
ORs	Odds ratios
CIs	Confidence intervals
