# Supplementary material for: Revisiting Thyroid Function in Patients Undergoing Electroconvulsive Therapy for Severe or Treatment-Resistant Depression
Source: J Clin Med. 2026 Feb 25;15(5):1740. doi: 10.3390/jcm15051740 (PMC12986223; doi:10.3390/jcm15051740)
Supplement: Supplementary file 1 [file jcm-15-01740-s001.zip › jcm-4141397-supplementary.pdf]

Supplementary Material

Page:

2 **Table S1.** Logistic regression analysis, Dependent variable: Response

3 **Table S2.** Correlation matrix (n=76)

4 **Figure S1.** Flow-diagram of the sample. ECT: Electroconvulsive therapy, TSH: Thyrotropin

5 **Figure S2.** Distribution of Thyrotropin (TSH) levels of the sample

6 **Figure S3.** Dendrogram for patient clustering according to response status, diagnosis, psychotic features, sex and age.

Table S1. Logistic regression analysis, Dependent variable: Response.

Factor	Odds ratio (95% CI)	p
Age	1.014 (0.975-1.055)	0.494
Suicidality	1.779 (0.423-7.483)	0.432
Total days of hospitalization	0.975 (0.948-1.004)	0.093
Number of unsuccessful treatment trials	0.859 (0.590-1.249)	0.425
HAMD score before ECT	1.098 (0.977-1.233)	0.116
Free-T4 levels	1.016 (0.795-1.299)	0.896
Model $\chi2$=3.785; p=0.876		

$\chi2$: Chi-square, ECT: Electroconvulsive therapy, HAMD: Hamilton Depression Rating Scale, Free-T4: free thyroxine

Table S2. Correlation matrix (n=76).

		TSH	FT3	FT4	Sex 1: female, 2: male	Age	Diagnosis 1: Bipolar disorder 2: Major depression	Psychotic features	Suicidality	HAMD before ECT	HAMD after ECT	Percentage change in HAMD	Number of ECT sessions	Duration of the index episode
TSH	Coefficient	1.000	0.069	**-0.450***	-0.096	-0.153	-0.065	-0.173	0.098	-0.203	0.060	-0.143	0.046	0.186
	p	.	0.551	**<0.001**	0.408	0.187	0.577	0.134	0.401	0.079	0.604	0.218	0.691	0.108
FT3	Coefficient		1.000	-0.151	0.150	**-0.421***	0.062	**-0.378***	**0.239***	-0.199	0.114	-0.202	-0.215	0.157
	p		.	0.194	0.195	**<0.001**	0.592	**0.001**	**0.038**	0.085	0.328	0.081	0.062	0.175
FT4	Coefficient			1.000	-0.033	0.204	-0.019	0.194	-0.087	**0.397***	0.015	0.144	-0.188	-0.198
	p			.	0.776	0.078	0.872	0.092	0.455	**<0.001**	0.895	0.216	0.105	0.087
Sex 1: female, 2: male	Coefficient				1.000	-0.026	0.017	0.137	0.086	-0.047	-0.181	0.171	0.069	**-0.259***
	p				.	0.821	0.881	0.236	0.458	0.689	0.118	0.141	0.552	**0.024**
Age	Coefficient					1.000	0.175	**0.426***	**-0.460***	0.082	-0.140	0.194	-0.007	-0.109
	p					.	0.130	**<0.001**	**<0.001**	0.482	0.227	0.093	0.955	0.349
Diagnosis 1: Bipolar disorder 2: Major depression	Coefficient						1.000	0.070	-0.013	0.017	0.224	-0.196	0.097	**0.278***
	p						.	0.548	0.910	0.881	0.051	0.090	0.407	**0.015**
Psychotic features	Coefficient							1.000	-0.191	0.131	-0.098	0.150	-0.047	**-0.247***
	p							.	0.098	0.259	0.401	0.195	0.689	**0.032**
Suicidality	Coefficient								1.000	-0.035	0.128	-0.148	0.010	0.129
	p								.	0.761	0.269	0.201	0.934	0.267
HAMD before ECT	Coefficient									1.000	0.159	0.204	0.159	-0.134
	p									.	0.170	0.077	0.170	0.247
HAMD after ECT	Coefficient										1.000	**-0.920***	**0.314***	**0.275***
	p										.	**<0.001**	**0.006**	**0.016**
Percentage change in HAMD	Coefficient											1.000	**-0.253***	**-0.336***
	p											.	**0.027**	**0.003**

Number of ECT sessions	Coefficient									1.000	**0.229***
	p									.	**0.047**
Duration of the index episode	Coefficient										1.000
	p										.

ECT: Electroconvulsive therapy, HAMD: Hamilton Depression Rating Scale, TSH: Thyrotropin, FT3: free triiodothyronine, FT4: free thyroxine. **Bold** cells indicate statistical significance.

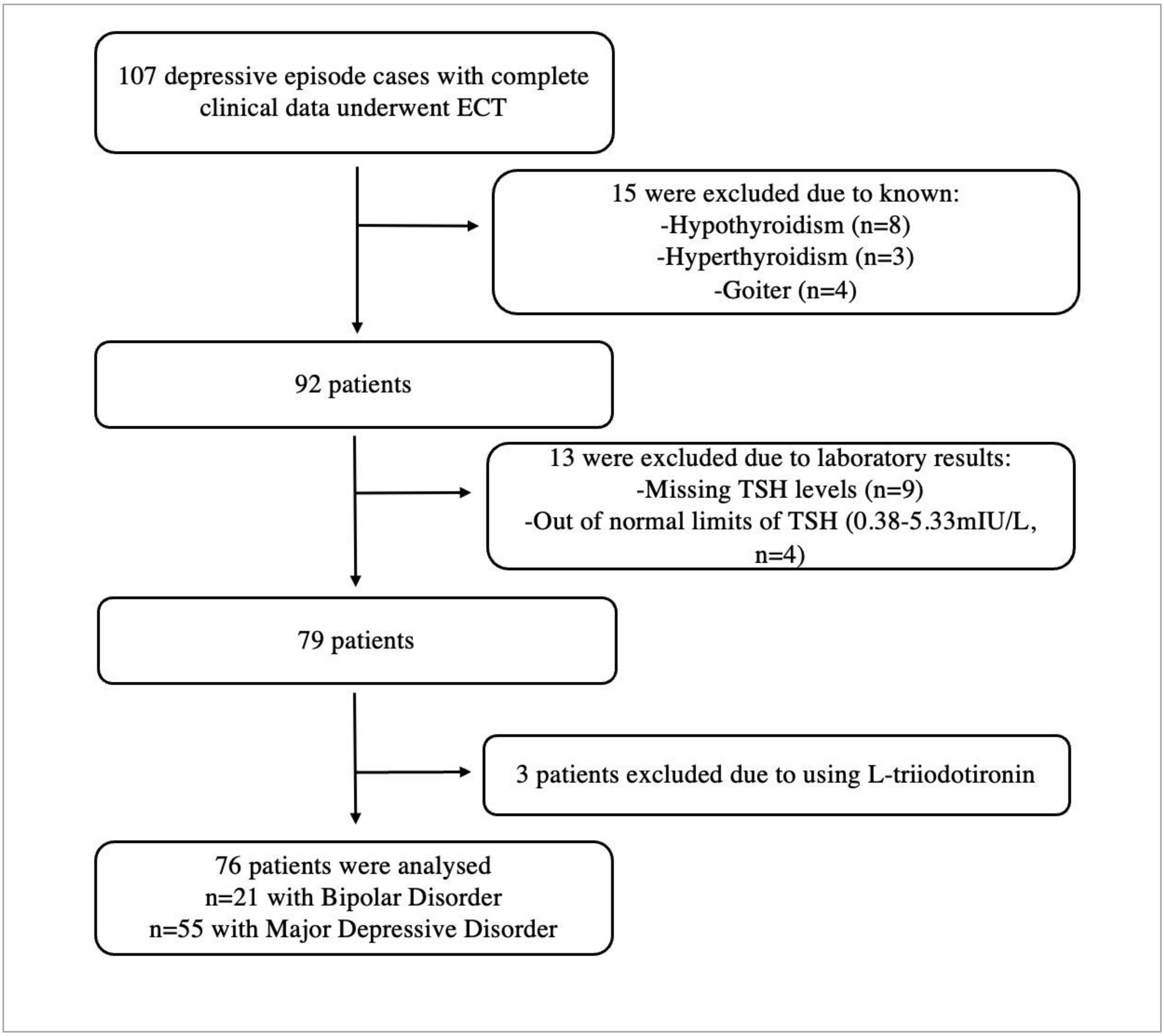

Figure S1. Flow-diagram of the sample. ECT: Electroconvulsive therapy, TSH: Thyrotropin

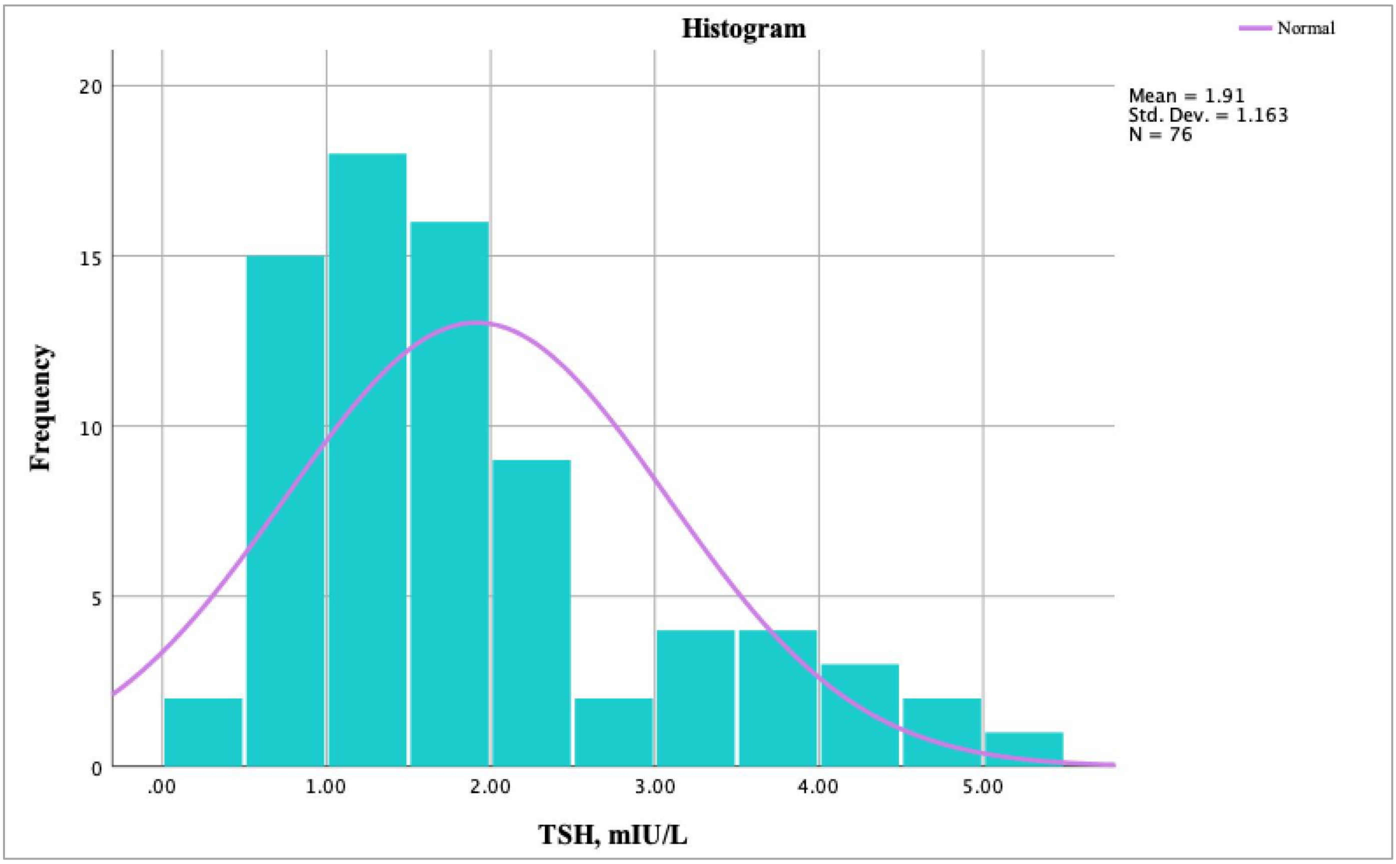

Figure S2. Distribution of Thyrotropin (TSH) levels of the sample

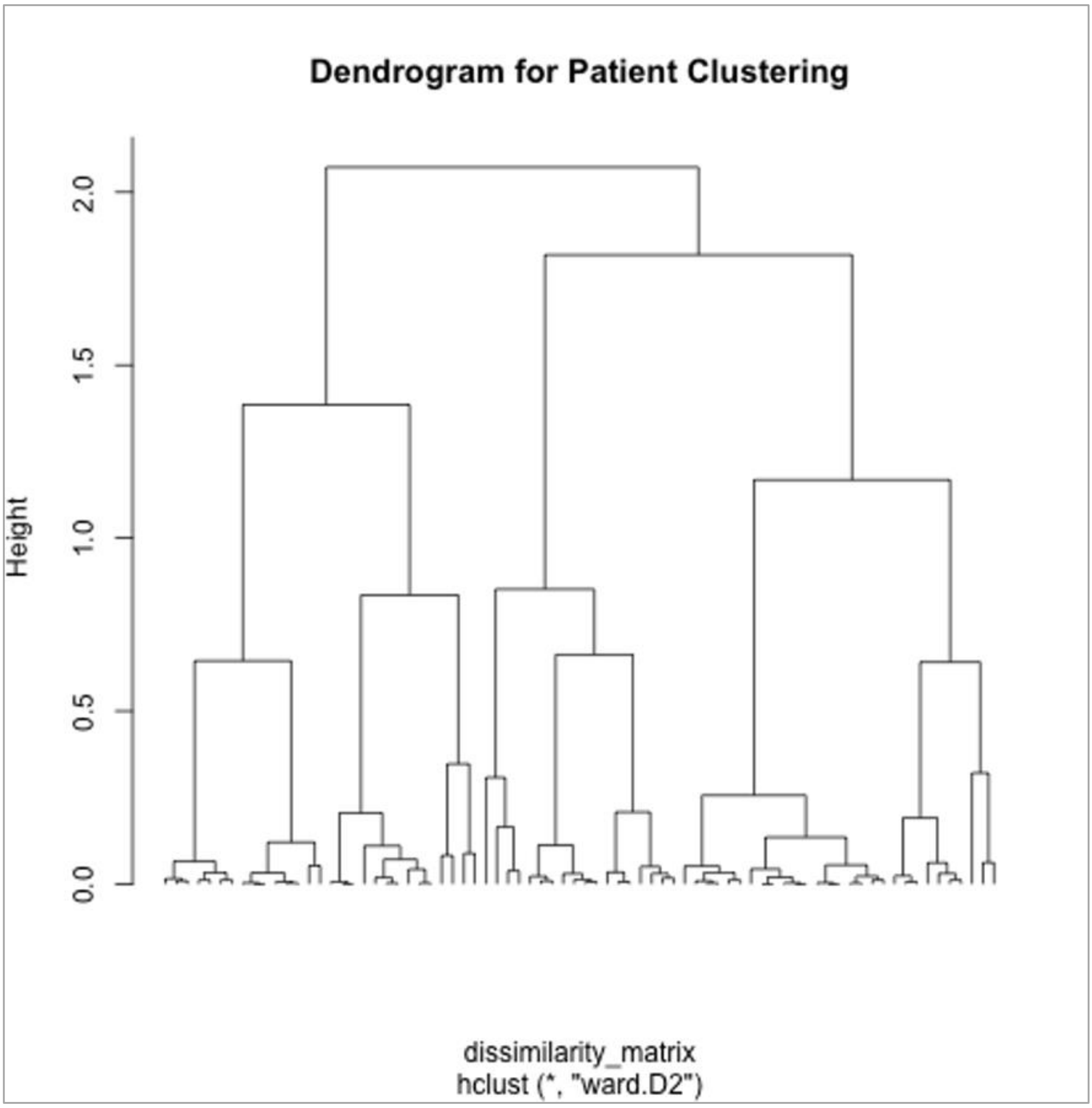

Figure S3. Dendrogram for patient clustering according to response status, diagnosis, psychotic features, sex and age.
